# Alexander view as a stand-alone alternative to standard anteroposterior imaging for treatment decisions in acute acromioclavicular joint dislocation

**DOI:** 10.1016/j.jseint.2026.101726

**Published:** 2026-04-30

**Authors:** Leopold Henssler, Luis Huber, Lisa Klute, Jonas Krueckel, Andreas Voss, Volker Alt, Maximilian Kerschbaum

**Affiliations:** a University Hospital Regensburg, Department of Trauma Surgery, Regensburg, Germany; b Sporthopaedicum, Regensburg, Germany

**Keywords:** Acromioclavicular joint, Alexander view, Horizontal instability, Rockwood classification, Circle measurement, Shoulder injury

## Abstract

**Background:**

To determine whether treatment decision-making in acromioclavicular joint injuries is possible using only Alexander views with diagnostic reliability comparable to conventional pre-operative imaging.

**Methods:**

Patients with acute first-time acromioclavicular joint injuries (Rockwood II-V) at 2 specialized centers (January 2019-June 2024) were retrospectively reviewed. Inclusion required complete pre-operative imaging consisting of either a panoramic view or bilateral Zanca views, plus at least one pre-operative modified Alexander stress view. A reference classification was established using coracoclavicular distance measurements, supplemented by the modified Alexander projection. Patients were stratified into a nonoperative group (Rockwood II-IIIA) and a potentially operative group (Rockwood IIIB-V). Modified circle measurements were performed for Alexander views. Three independent surgeons first assessed all cases using anteroposterior radiographs plus Alexander view and again after a 2-week washout period using only the Alexander view and recommended operative or nonoperative treatment. Inter-rater and intrarater reliability, diagnostic accuracy relative to the reference classification, and the correlation between circles values and injury severity were calculated. Receiver operating characteristic analyses were conducted to determine cutoff values for distinguishing higher-grade injuries.

**Results:**

54 patients met inclusion criteria. Using complete imaging, raters demonstrated a sensitivity of 95.1% and specificity of 90.1% for treatment allocation with substantial inter-rater agreement (κ = 0.653). Using only Alexander views, diagnostic performance remained high (sensitivity 87.7%, specificity 90.1%) with substantial inter-rater (κ = 0.753) and substantial to almost perfect intrarater reliability (κ = 0.779-0.852). Modified circles values correlated strongly with injury severity (τ = 0.753, *P* < .001). An 18-mm threshold differentiated Rockwood IIIB from V injuries with high sensitivity (92.9%) and negative predictive value (97.2%).

**Conclusion:**

The Alexander view alone enabled accurate identification of high-grade instability as defined by Rockwood's classification and supported treatment decisions comparable to standard anteroposterior imaging. These findings suggest that routine panorama or Zanca views may not be required for decision-making in acute AC joint injuries.

Acute acromioclavicular (AC) joint dislocations are common injuries in young, active individuals^8^^,^^14^ and present with varying degrees of vertical and horizontal instability.^14^ Treatment selection remains largely guided by imaging findings in combination with patient-specific factors.^4^ Conventional radiographic assessment depends on weighted bilateral anteroposterior (AP) panorama views,^25^ as current treatment algorithms are based on the Rockwood classification,^3^^,^^4^^,^^25^ which requires side-to-side comparison of coracoclavicular (CC) distances. This bilateral measurement of vertical displacement is central to differentiating low-grade injuries that can be treated nonoperatively^28^ from high-grade injuries where surgical intervention is indicated.^3^^,^^4^

However, widely used panorama views have clear limitations. They cannot assess horizontal instability, which influences clinical outcome^16^ since horizontal instability has shown inferior clinical results with nonoperative treatment. Although the Rockwood classification, based on CC distances, correlates with CC ligament disruption, injury severity is frequently misjudged. Nearly half of cases are misclassified when compared with magnetic resonance imaging findings.^19^ There is also uncertainty whether weighted stress views are necessary in all patients.^3^^,^^21^^,^^22^ In addition, this imaging modality is vulnerable to technical variations, particularly in tilt and rotation, that may alter radiographic projections, potentially leading to misinterpretation of injury severity and influencing treatment decisions.^10^ Moreover, it exposes vulnerable structures such as the thyroid gland, the lungs, and the esophagus to radiation despite these organs not being the subject of evaluation. To reduce radiation exposure, bilateral Zanca views are regarded as an alternative.^25^ Although they provide focused AP imaging with lower dose, they require 2 separate exposures, making projection deviation between sides almost unavoidable, which reduces reliability of CC distance measurements. Moreover, like panorama views, Zanca views also cannot capture horizontal translation.

In recent years, these limitations have shifted attention toward a modified Alexander projection.^2^ In contrast to the original description,^2^ a modified lateral radiograph of the AC joint which is obtained with the shoulder thrust anteriorly and the arm in cross-body adduction to stress the joint,^18^ combined with a slight cranial beam tilt (approximately 10°), has shown helpful in assessment of both vertical and horizontal instability in one image. This projection has shown high sensitivity for detecting Rockwood type III and V injuries,^5^ which are the patterns most frequently associated with indications for surgical management.^4^ Quantitative parameters obtained from this projection, including the acromial center line to dorsal clavicle and glenoid center line to posterior clavicle distances, show excellent reliability and strong construct validity and are largely unaffected by minor malrotation.^32^ However, the complexity of these measurements limits their routine use in everyday clinical practice. The recently introduced circle measurement^20^ offers a simpler, direction-independent alternative with excellent reliability and robust convergent and discriminant validity. Clinical studies confirm that circle values correlate closely with CC distance, reliably distinguish between Rockwood IIIA and IIIB injuries, reflect the degree of dynamic horizontal translation (DHT) in vivo,^30^ and are useful in assessment of treatment results as they correlate with post-operative outcomes.^17^^,^^29^

Because current guidelines consider any operative management only in the presence of horizontal translation (Rockwood IIIB and V), axial or Alexander views are frequently obtained in addition to standard AP radiographs to assess dynamic horizontal instability. Therefore, Alexander views are the only radiographic modality from which a direct therapeutic conclusion can be drawn. Consequently, the aim of this study was to determine whether treatment decisions can also be derived from the Alexander view alone, thereby eliminating the need for additional panorama or Zanca imaging.

## Methods

### Patient cohort

Patients presenting with acute AC joint injuries at one level I trauma center and one specialized orthopedic sports medicine center between January 2019 and June 2024 were retrospectively identified. Medical and radiographic records were screened, and only primary, first-time AC joint injuries classified as Rockwood type II-V were eligible for inclusion. Additional inclusion criteria required complete pre-operative imaging consisting of either a weighted panoramic stress view or bilateral Zanca views and at least one Alexander view of the affected shoulder. Exclusion criteria included age less than 18 years, any history of prior AC joint injury or surgery, incomplete or technically inadequate imaging, and missing clinical data.

### Radiographic imaging

All patients underwent comprehensive imaging of the AC joint. Since only including acute injuries, standard AP imaging of both AC joints was performed using either Zanca views or unweighted panoramic stress views, as weighted views have not been shown to alter classification compared with unweighted images.^21^ In addition, at least one modified Alexander projection of the injured AC joint was obtained using the technique described by Minkus et al.^18^ For this modified lateral radiograph, the patient is positioned at approximately 45° to the detector, similar to a shoulder Y-view. The injured arm is placed in cross-body adduction with the hand resting on the contralateral shoulder to increase stress across the AC joint. In this position, the scapula is oriented parallel to the sagittal plane and displaced away from the thoracic cage.

### Radiographic assessment

**Figure 1:**
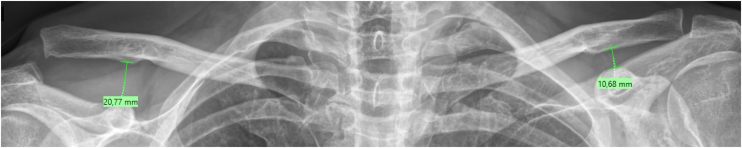
Example of a panoramic view of an acute injury to the acromioclavicular joint with a 94.5% increase in coracoclavicular distance, corresponding to a Rockwood type III injury.

In the first step, all eligible cases were pseudonymized and sorted in random order using lists of random numbers for further processing. All injuries were then classified according to the Rockwood system^24^ by an experienced, board-certified shoulder surgeon. Classification was based on CC distance measurements (Fig. 1) obtained from panorama views or bilateral Zanca views. Type III was divided into stable (IIIA) and unstable (IIIB) injuries according to the International Society of Arthroscopy, Knee Surgery & Orthopaedic Sports Medicine.^3^ The International Society of Arthroscopy, Knee Surgery & Orthopedic Sports Medicine defines unstable type IIIB clinically as a treatment-resistant dysfunction of the scapula and/or radiologically as an overlap of the lateral clavicle on Alexander images. In line with the recent research^20^^,^^30^ on the radiological diagnosis of AC joint injuries, the present study focused on differentiation based on radiological aspects and used the Alexander view of the affected shoulder for the discrimination of Rockwood types IIIA and IIIB.

In accordance with current treatment guidelines, injuries were stratified into a primarily nonoperative group (Rockwood I-IIIA) and a potentially operative group (Rockwood IIIB-V). This stratification served as the reference standard for all subsequent analyses.

**Figure 2:**
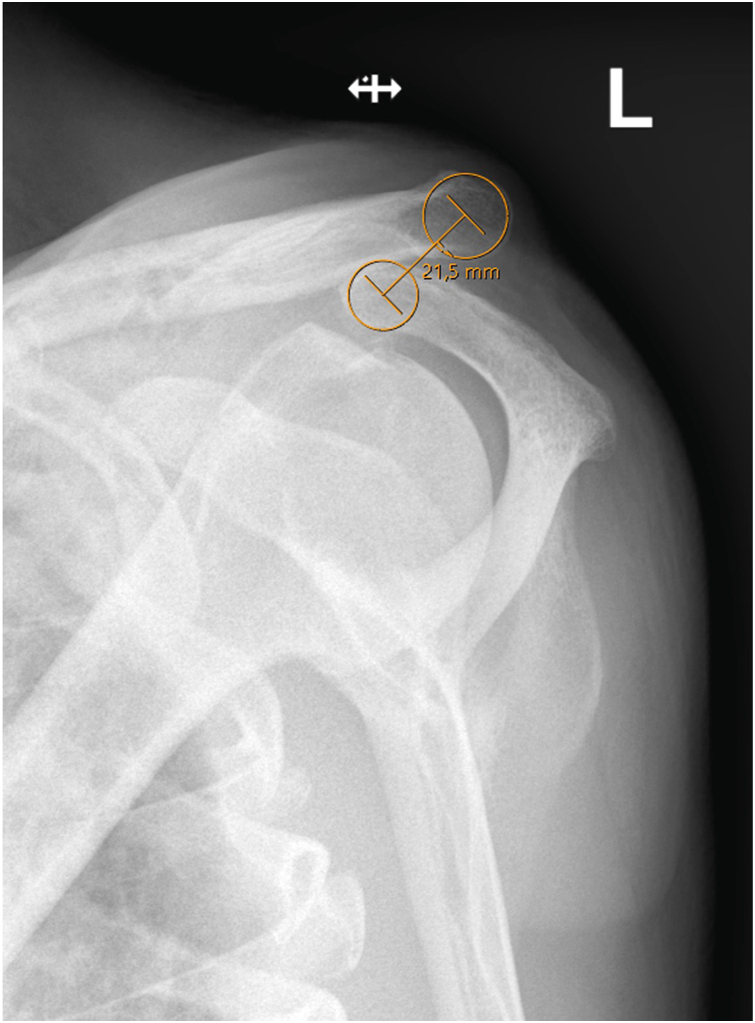
Example of a modified circle measurement on an Alexander view of an acutely injured acromioclavicular joint demonstrating vertical and dynamic horizontal instability. For this assessment, a *circle* is drawn to encompass the lateral contour of the clavicle, and another *circle* is drawn around the anteromedial aspect of the acromion. The distance between the centers of these 2 circles is then measured. In contrast to the originally described technique, the present study applied a modified approach in which the measurement was performed exclusively on the injured side.

DHT was additionally assessed using the criteria described by Kraus,^16^ and a modified circle measurement was performed as previously established.^20^^,^^30^ DHT was classified into 3 categories: no, partial, and complete horizontal translation. Absence of horizontal translation was defined by alignment of the clavicle with the acromion, without relevant posterosuperior displacement. Partial horizontal translation was defined as posterosuperior displacement of less than one clavicle width. Complete horizontal translation was defined as posterior displacement of one clavicle width or more. In contrast to the originally described techniques,^16^^,^^20^^,^^30^ the present study applied a modified approach in which the measurement was performed exclusively on the injured side (Fig. 2).

The eligible pseudonymized injuries were then presented to 3 additional independent shoulder surgeons and evaluated on the basis of all available standard AP radiographs together with the Alexander view. Each surgeon assigned a Rockwood grade, assessed the presence of dynamic horizontal instability, and recommended either nonoperative or operative treatment based on their assessment.

Following a 2-week washout period, the same raters reassessed all cases in another randomized order using only the Alexander view of the affected shoulder. During this second evaluation, raters were provided with the imaging data only and asked solely to determine whether nonoperative or operative treatment was indicated. At this stage, the raters were not given predefined quantitative criteria or cutoff values to guide their decision-making. Their task was to determine the appropriate treatment (operative vs. nonoperative) based on their subjective clinical assessment of vertical and horizontal instability as visualized in the Alexander view. Rockwood grading was not assessed in this round.

### Statistical analysis

Statistical analyses were performed using IBM SPSS Statistics (version 29; IBM Inc., Armonk, NY, USA), with statistical significance defined as *P* < .05. Continuous variables were analyzed and reported as mean ± standard deviation, whereas categorical variables were summarized as counts and percentages. Inter-rater and intrarater reliability for the surgeons' assessments was calculated using Fleiss' kappa. Diagnostic accuracy relative to the reference classification was determined by calculating sensitivity, specificity, and positive and negative predictive values. Kendall's tau was applied to evaluate the correlation between modified circle measurements and Rockwood types. Differences in circle distances across injury types were assessed using the Mann-Whitney U test. Receiver operating characteristic analysis was used to determine the optimal cutoff value of the circles distance for distinguishing Rockwood type IIIB from type V injuries.

## Results

### Patient cohort

A total of 75 patients with acute AC joint injuries were initially screened. After application of the inclusion and exclusion criteria, 17 patients were excluded for incomplete imaging, 3 patients for incomplete clinical data, and one patient below the age of 18 years. Consequently, 54 patients remained eligible for final analysis. The mean patient age was 39.9 ± 14.7 years, and the majority of patients were male (n = 43; 79.6%). Based on the reference classification, 18 patients (33.3%) were identified as Rockwood type II, 9 patients (16.7%) as type IIIA, 13 patients (24.1%) as type IIIB, and 14 patients (25.9%) as type V. This resulted in an equal distribution between patients with and without a surgical indication (27/54 each).

### Raters' allocation to treatment groups

In the initial assessment using complete imaging, the 3 raters demonstrated substantial inter-rater reliability for Rockwood classification (κ = 0.653). No major misclassifications occurred between clearly distinct injury severities. However, some variability persisted in differentiating intermediate-grade injuries, particularly between Rockwood types IIIA and IIIB (Table I).

**Table I:** The 3 raters’ assignments (3 raters × 54 patients = 162 assessments) to the Rockwood classification and their consistency with the reference assignment

Rockwood type (reference)	Rockwood type (raters)				Total
	II	IIIA	IIIB	V	
II	46	6	2	0	54
IIIA	3	18	6	0	27
IIIB	1	2	28	8	39
V	0	1	4	37	42
Total	50	27	40	45	162

Using standard imaging (AP radiographs plus an Alexander view), the raters classified patients into operative and nonoperative groups according to current guideline thresholds with a specificity of 90.1% and a sensitivity of 95.1% (Table II). Inter-rater agreement for this treatment allocation was substantial (Fleiss' κ = 0.747).

**Table II:** The 3 raters’ recommendations (3 raters × 54 patients = 162 assessments) based on standard imaging, consisting of anteroposterior (panoramic or bilateral Zanca view + Alexander view)

Rockwood type (reference)	Recommendation based on anteroposterior + Alexander view		Total
	Nonoperative	Consider surgery	
Nonoperative (Rockwood II & IIIA)	73	8	81
Consider surgery (Rockwood IIIB & V)	4	77	81
Total	77	85	162

When assessments were repeated using only the Alexander view, diagnostic accuracy remained high, with a specificity of 90.1% and a sensitivity of 87.7% (Table III). Inter-rater reliability was similar to the full imaging assessment (κ = 0.753), while intrarater reliability ranged from 0.779 to 0.852, reflecting substantial to almost perfect agreement.

**Table III:** The recommendations (3 raters × 54 patients = 162 assessments) made by the raters based solely on the Alexander view

Rockwood type (reference)	Recommendation based on Alexander view only		Total
	Nonoperative	Consider surgery	
Nonoperative (Rockwood II & IIIA)	73	8	81
Consider surgery (Rockwood IIIB & V)	10	71	81
Total	83	79	162

### Modified circle measurements

To improve objectivity in treatment decisions, circle distances were further analyzed and demonstrated a significant correlation between the modified circle measurement and injury severity according to the Rockwood classification (τ = 0.753, *P* < .001). Circle distances increased progressively with higher Rockwood grades, with a significant difference between types IIIB and V (*P* = .001). Although the difference between types IIIA and IIIB did not reach statistical significance (*P* = .095), circle distances differed significantly between the nonoperative group (Rockwood II and IIIA) and the operative group (Rockwood IIIB and V) (*P* < .001).

However, receiver operating characteristic analysis identified an optimal cutoff of 15.9 mm to distinguish high-grade instability requiring operative treatment (Rockwood IIIB and V) from lower-grade injuries (Rockwood II and IIIA), with an AUC of 0.924. This cutoff yielded a sensitivity of 70.4%, specificity of 96.3%, positive predictive value of 95.0%, and negative predictive value of 76.5%.

**Figure 3:**
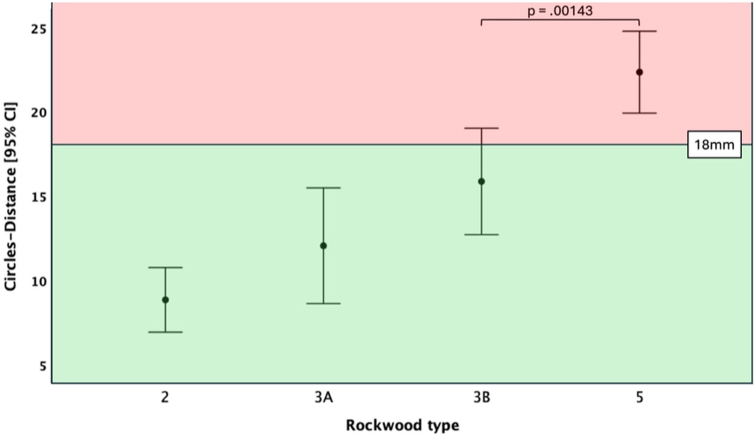
Mean circle measurements *(dots)* for each Rockwood type with 95% confidence intervals (95% CIs; whiskers). A modified unilateral circles distance of 18 mm was identified through receiver operating characteristic analysis as the optimal threshold for distinguishing between Rockwood types IIIB and V.

For differentiating the highest grade (Rockwood type V) injuries from lower-grade patterns (Fig. 3), the optimal cutoff was 18.0 mm (AUC 0.929), with a sensitivity of 92.9%, specificity of 87.5%, positive predictive value of 72.2%, and negative predictive value of 97.2%.

## Discussion

The purpose of this study was to determine whether treatment decisions for acute AC joint injuries can be derived from the Alexander view alone with diagnostic reliability comparable to the current standard of pre-operative imaging.

The principal finding of this study is that high-grade, combined biplanar instability can be identified with high diagnostic accuracy using the Alexander projection alone. This allows treatment decisions in line with current guidelines without the need for bilateral panorama or Zanca radiographs. When injuries were assessed using only Alexander views, sensitivity, specificity, and inter-rater agreement were comparable to evaluations based on full standard imaging, including bilateral CC distance measurements. Moreover, the modified circle measurement further proved helpful in clinical decision-making by distinguishing patients with an indication for surgery (Rockwood types IIIB and V) from those suitable for conservative treatment (Rockwood types II and IIIA). A circle distance of more than 16 mm was found in 95% of all Rockwood type IIIB or V injuries. Among patients with a relative indication for surgery, the modified circle method also enabled further subclassification of horizontally unstable injuries. A unilateral circle distance >18 mm identified Rockwood type V injuries in 92.9% of cases, while values below this threshold effectively excluded type V instability in 97.2% of cases. This distinction is clinically relevant, as Rockwood IIIB injuries may initially be managed nonoperatively, with outcomes comparable to surgical treatment in the mid-term.^13^^,^^27^ These findings collectively suggest that the Alexander view may serve as a sufficient standalone radiographic modality for treatment decision-making, particularly because horizontal instability can be reliably quantified using this projection.

Traditional imaging strategies for evaluating AC joint injuries have centered on bilateral comparison of the CC distance, following the principles of the original Rockwood classification. Panorama views remain the recommended modality in most contemporary guidelines,^25^ supported by evidence demonstrating reliable grading of AC joint injuries with high interobserver and intraobserver consistency when CC distance measurements are used^26^ and a correlation between increasing CC distance and CC ligament disruption.^19^ Nevertheless, these imaging concepts exhibit important limitations. Standard AP projections do not capture horizontal instability, and prior work has shown considerable misclassification when Rockwood grades are compared with magnetic resonance imaging–based assessments of ligament integrity.^19^ In recent years, the clinical relevance of the Rockwood classification has increasingly been questioned. Several studies have demonstrated weak correlation between Rockwood grade and patient-reported symptoms.^11^ Moreover, randomized controlled trials have challenged the long-held assumption that higher-grade injuries warrant primary surgical management. Multiple trials reported no functional superiority of operative treatment over nonoperative care for Rockwood III injuries^6^^,^^7^^,^^27^ and even for Rockwood V lesions,^1^^,^^6^^,^^7^ particularly beyond mid-term follow-up.^15^ These findings must be interpreted with caution, however, as many studies grouped Rockwood III and V injuries together^6^^,^^7^^,^^15^ and frequently evaluated hook plate fixation,^6^^,^^15^^,^^27^ a technique now known to yield inferior outcomes compared with modern suspensory suture-button constructs.^31^ Despite these limitations, an initial nonoperative regimen with interval reassessment at 6 weeks has been proposed even for higher-grade injuries. This strategy allows identification of patients with persistent pain or restricted motion who may subsequently benefit from surgical stabilization.^12^ While this staged approach has been incorporated into current recommendations for Rockwood IIIB injuries, it has not yet been broadly adopted for Rockwood V lesions, which remain widely considered indications for primary surgical intervention^4^ due to less favorable outcomes observed with nonoperative management.^9^

Because horizontal translation cannot be reliably assessed using bilateral AP imaging alone, the Alexander view has gained increasing importance in the diagnostic workup of acute AC joint injuries. The present findings are consistent with growing evidence supporting lateral-based radiographic assessment, as axial imaging has demonstrated limited specificity for detecting horizontal instability in cadaveric analyses.^23^ Beyond offering a potentially safer, lower-radiation alternative, the principal clinical value of the Alexander projection lies in its improved visualization of horizontal instability, a key factor in distinguishing clinically relevant injury patterns. Therefore, it enables reliable assessment of both AP and vertical translation and demonstrates strong interobserver measurement consistency.^2^^,^^20^^,^^29^^,^^32^

Likewise, the recently introduced circle measurement has shown strong correlation with vertical displacement^20^^,^^30^ and the ability to distinguish Rockwood IIIA from IIIB injuries in vivo.^30^ In the present study, cases qualifying for surgery (both Rockwood IIIB and V) could be identified with high diagnostic accuracy based on the subjective assessment of combined high-grade vertical and horizontal instability using the Alexander view alone. This highlights its potential to directly influence clinical decision-making by depicting both planes of instability in a single image rather than merely complementing conventional imaging.

In addition, the circle value proved to be clinically meaningful, showing a strong correlation with Rockwood grade and reliably distinguishing Rockwood IIIB from type V injuries using a cutoff value of 18 mm. This distinction is relevant, as current practice still generally favors surgical treatment for type V injuries, while type IIIB injuries can initially be managed nonoperatively, with close reassessment after several weeks. Although the cutoff value of 18 mm identified for differentiating between Rockwood IIIB and V injuries differs from previously reported thresholds,^30^ this discrepancy underscores the need for further validation of cutoff values in future clinical trials.

Overall, the present findings extend prior biomechanical^20^ and clinical evidence^29^^,^^30^ by demonstrating that Alexander-based assessment not only provides reliable measurements but also enables clinical real-world decision-making in a consecutive trauma population. From a practical perspective, the ability to identify patients requiring surgical intervention with high diagnostic accuracy using the Alexander view alone suggests that it could serve as a standalone imaging modality in selected settings, simplifying diagnostic workflows while maintaining decision accuracy. As with any stand-alone technique, its reliability depends on proper execution and image quality. Nonetheless, these findings question the necessity of routine bilateral AP imaging and, by extension, the reliance on the Rockwood classification for guiding initial management in acute AC joint instability.

Several limitations of this study should be acknowledged. First, the retrospective design carries an inherent risk of selection bias, particularly because approximately one-third of the initially screened patients were excluded due to incomplete imaging. Second, the reference standard was based on the Rockwood classification, which is susceptible to projectional variability and may not fully reflect the true extent of ligamentous injury, although this classification remains the basis for treatment decisions in routine clinical practice. Another limitation is that the study was conducted at 2 specialized centers with surgeons experienced in AC joint imaging. Therefore, diagnostic performance might differ in lower-volume settings. Finally, prospective validation of the study results and assessment of clinical outcomes are necessary before routine abandonment of bilateral AP imaging can be recommended.

## Conclusion

This study supports a simplified imaging strategy for the evaluation of treatment decisions in acute AC joint injuries, highlighting the Alexander view as a key diagnostic projection for guiding treatment. By enabling simultaneous assessment of vertical and horizontal instability, the Alexander view demonstrated accuracy of treatment categorization comparable to standard imaging in this cohort, indicating that it may serve as a more efficient and functionally meaningful modality than traditional bilateral AP techniques. The present findings suggest that once an AC joint injury is confirmed, associated injuries of the proximal humerus, scapula, and coracoid process are excluded, and an Alexander view is obtained, additional AP radiographs provide limited value in determining whether operative or nonoperative management is indicated. Consequently, these results question the continued necessity of panorama or bilateral Zanca views in the initial diagnostic workup of acute AC joint injuries. Further prospective studies are warranted to confirm these results and investigate their impact on clinical decision-making and patient-reported outcomes.

## Declaration of Generative AI and AI-Assisted Technologies in the Writing Process

During the preparation of this work the authors used ChatGPT (GPT-5.1) in order to check for spelling and grammar and improve readability. After using this tool/service, the authors reviewed and edited the content as needed and take full responsibility for the content of the publication.

## Disclaimers:

Funding: No funding was disclosed by the authors.

Conflicts of interest: The authors, their immediate families, and any research foundations with which they are affiliated have not received any financial payments or other benefits from any commercial entity related to the subject of this article.

## References

[1] Akgün D., Gebauer H., Paksoy A., Eckl L., Hayta A., Ücertas A. (2024). Comparison of clinical outcomes between nonoperative treatment and arthroscopically assisted stabilization in patients with acute rockwood type 5 acromioclavicular dislocation. Orthop J Sports Med.

[2] Alexander O.M. (1949). Dislocation of the acromioclavicular joint. Radiography.

[3] Beitzel K., Mazzocca A.D., Bak K., Itoi E., Kibler W.B., Mirzayan R. (2014). ISAKOS Upper Extremity Committee consensus statement on the need for diversification of the rockwood classification for acromioclavicular joint injuries. Arthroscopy.

[4] Berthold D.P., Muench L.N., Dyrna F., Mazzocca A.D., Garvin P., Voss A. (2022). Current concepts in acromioclavicular joint (AC) instability – a proposed treatment algorithm for acute and chronic AC-joint surgery. BMC Musculoskelet Disord.

[5] Bezruchenko S., Dolhopolov O., Yarova M., Luchko R., Mazevych V. (2022). Clinical evaluation and instrumental diagnostics in acute acromioclavicular joint dislocation. Ortop Traumatol Rehabil.

[6] Boström Windhamre H., von Heideken J., Une-Larsson V., Ekström W., Ekelund A. (2022). No difference in clinical outcome at 2-year follow-up in patients with type III and V acromioclavicular joint dislocation treated with hook plate or physiotherapy: a randomized controlled trial. J Shoulder Elbow Surg.

[7] Canadian Orthopaedic Trauma Society (2015). Multicenter randomized clinical trial of nonoperative versus operative treatment of acute Acromio-Clavicular joint dislocation. J Orthop Trauma.

[8] Chillemi C., Franceschini V., Dei Giudici L., Alibardi A., Salate Santone F., Ramos Alday L.J. (2013). Epidemiology of isolated acromioclavicular joint dislocation. Emerg Med Int.

[9] Dunphy T.R., Damodar D., Heckmann N.D., Sivasundaram L., Omid R., Hatch G.F. (2016). Functional outcomes of type V acromioclavicular injuries with nonsurgical treatment. J Am Acad Orthop Surg.

[10] Frege S., Lacheta L., Karpinski K., Paksoy A., Akgun D., Thiele K. (2025 Apr). Influence of tilt and rotation on coracoclavicular distance measurements and rockwood classification in panorama view radiographs in the diagnosis of acromioclavicular dislocations. Orthop J Sports Med.

[11] Granville-Chapman J., Torrance E., Rashid A., Funk L. (2018). The Rockwood classification in acute acromioclavicular joint injury does not correlate with symptoms. J Orthop Surg (Hong Kong).

[12] Haugaard K.B., Bak K., Ryberg D., Muharemovic O., Hölmich P., Barfod K.W. (2024). Patient-reported, clinical and radiological factors associated with the result after non-surgical management of acute AC joint dislocation Rockwood type III and V. Knee Surg Sports Traumatol Arthrosc.

[13] Haugaard K.B., Bak K., Ryberg D., Muharemovic O., Hölmich P., Barfod K.W. (2024). The ISAKOS subclassification of Rockwood type III AC joint dislocations in a stable type A and an unstable type B is not clinically relevant. Knee Surg Sports Traumatol Arthrosc.

[14] Haugaard K.B., Bak K., Seem K., Hölmich P., Barfod K.W. (2023). Rockwood type III is the most common type of acromioclavicular joint dislocation: a prospective cohort study investigating the incidence and epidemiology of acute acromioclavicular joint dislocations in an urban population. Shoulder Elbow.

[15] Joukainen A., Kröger H., Niemitukia L., Mäkelä E.A., Väätäinen U. (2014). Results of operative and nonoperative treatment of rockwood types III and V acromioclavicular joint dislocation: a prospective, randomized trial with an 18- to 20-Year Follow-up. Orthop J Sports Med.

[16] Kraus N., Hann C., Gerhardt C., Scheibel M. (2018). Dynamic instability of the acromioclavicular joint: a new classification for acute AC joint separation. Obere Extrem.

[17] Maziak N., Audige L., Hann C., Minkus M., Scheibel M. (2019). Factors predicting the outcome after arthroscopically assisted stabilization of acute high-grade acromioclavicular joint dislocations. Am J Sports Med.

[18] Minkus M., Hann C., Scheibel M., Kraus N. (2017). Quantification of dynamic posterior translation in modified bilateral Alexander views and correlation with clinical and radiological parameters in patients with acute acromioclavicular joint instability. Arch Orthop Trauma Surg.

[19] Minkus M., Wieners G., Maziak N., Plachel F., Scheibel M., Kraus N. (2021). The ligamentous injury pattern in acute acromioclavicular dislocations and its impact on clinical and radiographic parameters. J Shoulder Elbow Surg.

[20] Murphy R.J., Moor B.K., Lesniewski P.J., Hayoz A., Alcantara W., Zumstein M.A. (2021). Evaluation of the circles measurement and the ABC classification of acromioclavicular joint injuries. Am J Sports Med.

[21] Nordin J.S., Mogianos F., Hauggaard A., Lunsjö K. (2021). Weighted or internal rotation radiographs are not useful in the classification of acromioclavicular joint dislocations. Acta Radiol.

[22] Pogorzelski J., Beitzel K., Ranuccio F., Wörtler K., Imhoff A.B., Millett P.J. (2017). The acutely injured acromioclavicular joint – which imaging modalities should be used for accurate diagnosis? A systematic review. BMC Musculoskelet Disord.

[23] Rahm S., Wieser K., Spross C., Vich M., Gerber C., Meyer D.C. (2013). Standard axillary radiographs of the shoulder may mimic posterior subluxation of the lateral end of the clavicle. J Orthop Trauma.

[24] Rockwood C.A., Green D.P. (1984). Fractures in adults.

[25] Rosso C., Martetschläger F., Saccomanno M.F., Voss A., Lacheta L., Beitzel K., ESA DELPHI Consensus Panel (2021). High degree of consensus achieved regarding diagnosis and treatment of acromioclavicular joint instability among ESA-ESSKA members. Knee Surg Sports Traumatol Arthrosc.

[26] Schneider M.M., Balke M., Koenen P., Fröhlich M., Wafaisade A., Bouillon B. (2016). Inter- and intraobserver reliability of the Rockwood classification in acute acromioclavicular joint dislocations. Knee Surg Sports Traumatol Arthrosc.

[27] Tauber M., Hoffelner T., Lehmann L., Kraus N., Scheibel M., Moroder P. (2023). Prospective multicenter randomized controlled trial of surgical versus nonsurgical treatment for acute rockwood type 3 acromioclavicular injury. Orthopaedic J Sports Med.

[28] Verstift D.E., Kilsdonk I.D., van Wier M.F., Haverlag R., van den Bekerom M.P.J. (2021). Long-term outcome after nonoperative treatment for rockwood I and II acromioclavicular joint injuries. Am J Sports Med.

[29] Vetter P., Bellmann F., Eckl L., Paksoy A., Akgün D., Lazaridou A. (2025). The postoperative circles measurement considers recurrent instability and correlates with lower outcome scores after acute, bidirectional arthroscopically assisted acromioclavicular joint stabilization. Knee Surg Sports Traumatol Arthrosc.

[30] Vetter P., Eckl L., Bellmann F., Audigé L., Scheibel M. (2023). In vivo analysis of the circles measurement supports its use in evaluating acromioclavicular joint dislocations. J Shoulder Elbow Surg.

[31] Wang C., Meng J.-H., Zhang Y.-W., Shi M.-M. (2020). Suture button versus hook plate for acute unstable acromioclavicular joint dislocation: a meta-analysis. Am J Sports Med.

[32] Zumstein M.A., Schiessl P., Ambuehl B., Bolliger L., Weihs J., Maurer M.H. (2018). New quantitative radiographic parameters for vertical and horizontal instability in acromioclavicular joint dislocations. Knee Surg Sports Traumatol Arthrosc.

